# Crystal structure of the type IV secretion system component CagX from *Helicobacter pylori*

**DOI:** 10.1107/S2053230X17001376

**Published:** 2017-02-28

**Authors:** Jin Zhang, Fei Fan, Yanhe Zhao, Lifang Sun, Yadan Liu, Ronan M. Keegan, Michail N. Isupov, Yunkun Wu

**Affiliations:** a State Key Laboratory of Structural Chemistry, Fujian Institute of Research on the Structure of Matter, Chinese Academy of Science, Fuzhou 350002, People’s Republic of China; b Fujian Health College, Fuzhou 350101, People’s Republic of China; c CCP4, Research Complex at Harwell, Rutherford Appleton Laboratory, Harwell Oxford, Didcot OX11 0FA, England; d Institute of Integrative Biology, University of Liverpool, Liverpool L69 7ZB, England; e The Henry Wellcome Building for Biocatalysis, Biosciences, College of Life and Environmental Sciences, University of Exeter, Exeter EX4 4QD, England

**Keywords:** *Helicobacter pylori*, cytotoxin-associated gene pathogenicity island, *cag*PAI, type IV secretion system, CagX, crystal structure, X-ray diffraction

## Abstract

*Helicobacter pylori*, a Gram-negative bacterial pathogen prevalent in the human population, is the causative agent of severe gastric diseases. An *H. pylori* type IV secretion (T4S) system encoded by the cytotoxin-associated gene pathogenicity island (*cag*PAI) is responsible for communication with host cells. As a component of the *cag*PAI T4S system core complex, CagX plays an important role in virulence-protein translocation into the host cells. In this work, the crystal structure of the C-terminal domain of CagX (CagXct), which is a homologue of the VirB9 protein from the VirB/D4 T4S system, is presented. CagXct is only the second three-dimensional structure to be elucidated of a VirB9-like protein. Another homologue, TraO, which is encoded on the *Escherichia coli* conjugative plasmid pKM101, shares only 19% sequence identity with CagXct; however, there is a remarkable similarity in tertiary structure between these two β-sandwich protein domains. Most of the residues that are conserved between CagXct and TraO are located within the protein core and appear to be responsible for the preservation of this domain fold. The studies presented here will contribute to our understanding of different bacterial T4S systems.

## Abstract 2

The structure of the C-terminal domain of CagX is presented and structural comparisons with TraO, its homologue from another bacterial T4S system, reveal distinct and conserved features.

## 1. Introduction   

*Helicobacter pylori* is a Gram-negative bacterial pathogen which lives in the stomachs of more than half of the population of the world and causes chronic gastritis, peptic ulcers, gastric adenocarcinoma and gastric lymphoma (Blaser, 1997 ▸; Blaser & Atherton, 2004 ▸; Covacci *et al.*, 1999 ▸; Parsonnet *et al.*, 1994 ▸). The cytotoxin-associated gene pathogenicity island (*cag*PAI) contains a 40 kb foreign DNA region which is considered to be the main genetic determinant of more virulent *H. pylori* strains (Backert *et al.*, 2002 ▸; Blaser & Atherton, 2004 ▸). The *cag*PAI carries 27 genes which encode several effector proteins and one type IV secretion (T4S) system. T4S systems are important nanomachines in Gram-negative bacteria which play important roles in various biological processes from the transfer of virulence factors into eukaryotic cells to the conjugative delivery of genetic material and the uptake or release of DNA (Cascales & Christie, 2003 ▸). The T4S system of *H. pylori* translocates the major effector protein CagA into gastric epithelial cells. Subsequent phosphorylation of intracellular CagA leads to epithelial cell elongation and disruption of tight junctions, which results in the pathogenicity of *H. pylori* (Backert *et al.*, 2002 ▸; Blaser & Atherton, 2004 ▸; Odenbreit *et al.*, 2000 ▸; Segal *et al.*, 1997 ▸; Backert & Selbach, 2008 ▸). The T4S system has been visualized at the surface between *H. pylori* and gastric epithelial cells, forming a needle-like structure crossing the inner and outer membranes (Zanotti & Cendron, 2014 ▸). It has been proposed that the transmembrane core complex of the *H. pylori* *cag*PAI T4S system is formed by CagY, CagT and CagX, while the external pilus is thought to be composed of a large number of copies of CagC and CagL (Fischer, 2011 ▸; Terradot & Waksman, 2011 ▸).

As an essential component of the *cag*PAI T4S system core complex, CagX plays a critical role in CagA translocation into the host cell. Genetic and functional studies have indicated that the ability of *H. pylori* to translocate CagA into gastric cells is abrogated by inactivation of CagX (*hp0528*; Akopyants *et al.*, 1998 ▸; Censini *et al.*, 1996 ▸; Li *et al.*, 1999 ▸; Fischer *et al.*, 2001 ▸). The C-terminal domain of CagX has been shown to be responsible for its interaction with CagT by co-immuno­precipitation, MBP pull-down and yeast two-hybrid assays (Gopal *et al.*, 2015 ▸).

CagX is presumed to be structurally and functionally homologous to VirB9 from the well studied VirB/D4 T4S of the plant pathogen *Agrobacterium tumefaciens*, although the two proteins have low sequence identity (Bayliss *et al.*, 2007 ▸; Christie *et al.*, 2005 ▸; Censini *et al.*, 1996 ▸). While VirB9 forms a heterotrimer with VirB7 and VirB10 in the VirB/D4 T4S system, it is proposed that CagX associates not only with CagT and CagY, but also with CagM and Cagδ, to form a transmembrane core complex in the *cag*PAI T4S system (Kutter *et al.*, 2008 ▸; Pinto-Santini & Salama, 2009 ▸).

Here, we report the expression, purification, crystallization, structure determination and analysis of the C-terminal domain of the CagX protein (CagXct). This important component of the *cag*PAI T4S system folds into a β-sandwich domain containing nine β-strands. It is the first structure of a component of the transmembrane core complex of the *cag*PAI T4S system to be determined, and is only the second three-dimensional structure of a VirB9 homologue. The crystal structure was determined by the molecular-replacement method and refined at a resolution of 1.4 Å. Comparison of the CagXct structure with that of the C-terminal domain of another VirB9 homologue, TraO, which is a part of the outer membrane complex encoded by the *Escherichia coli* conjugative plasmid pKM101, suggests the possible conservation of some protein–protein interactions between the pKM101 T4S system and the *cag*PAI T4S system.

## 2. Materials and methods   

### 2.1. Cloning of expression constructs and protein expression   

**Figure 1:**
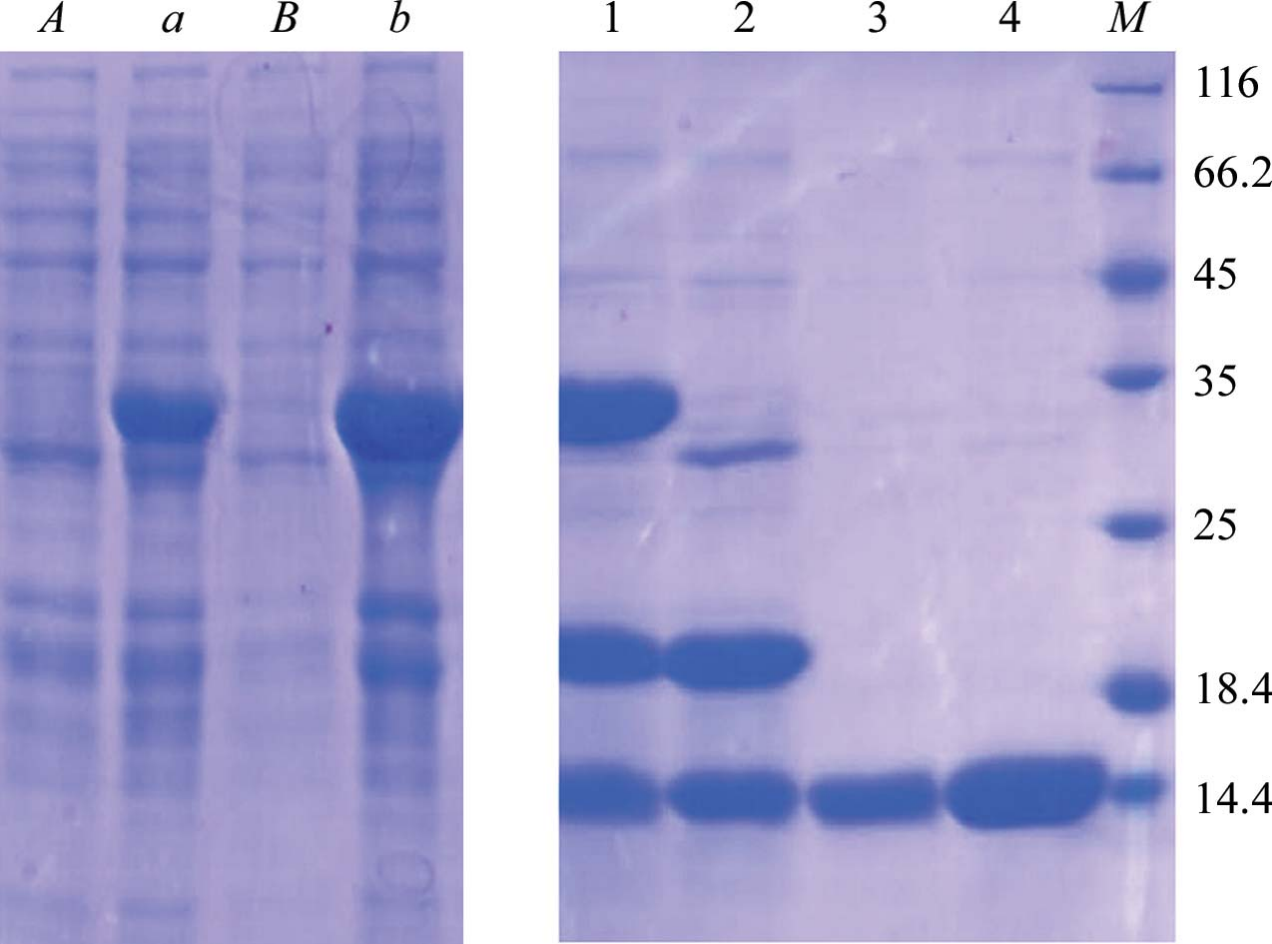
15% SDS–PAGE analysis of purified CagXct stained with Coomassie Brilliant Blue. Lane *A*, non-induced expression strains. Lane *a*, induced expression strains. Lane *B*, pellet fractions. Lane *b*, supernatant fractions. Lane 1, eluted with 500 m*M* imidazole; the band containing CagXct with a Trx tag is around 35 kDa. Lane 2, after cleavage by TEV; the band containing CagXct is under 14.4 kDa. Lane 3, flowthrough of the second nickel column. Lane 4, the sample before size-exclusion chromatography, showing high purity. Lane *M* contains molecular-mass marker (labelled in kDa).

The gene encoding the soluble fragment of Hp0528 (amino acids 396–498; CagXct) was amplified from *Hp26695* genomic DNA using the primer pair FCagXct/NCagXct and subcloned into pET-32a vector *via* EcoRI and XhoI (Table 1 ▸). The constructed pET-32a-CagXct was transformed into *E. coli* BL21 (DE3) cells. Expression of CagXct was performed in LB medium containing 100 µg ml^−1^ ampicillin and the culture was incubated at 37°C and 220 rev min^−1^ until an OD_600_ of 0.6–0.8 was reached. 0.3 m*M* isopropyl β-d-1-thiogalactopyranoside (IPTG) was added to induce the expression of recombinant CagXct and the culture was left to shake for 12 h at 16°C and 180 rev min^−1^. The expression levels of CagXct were monitored by SDS–PAGE (Fig. 1 ▸).

### 2.2. Protein purification and crystallization   

**Figure 2:**
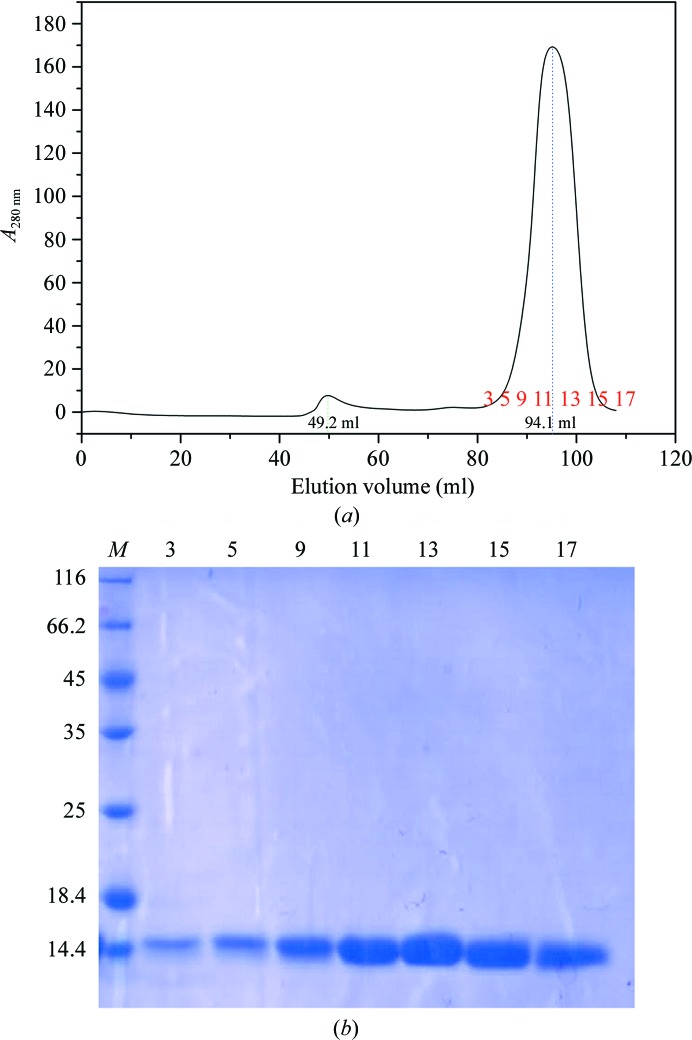
(*a*) Size-exclusion chromatography trace (HiLoad 16/600 Superdex 200 75 pg; GE Healthcare). CagXct elutes at 80–110 ml, which is in agreement with its monomer molecular mass of 11.4 kDa. The peak intensity is 192 mAU. Samples between 80 and 110 ml labelled 3–17 in red were picked for 15% SDS–PAGE analysis. (*b*) 15% SDS–PAGE analysis of the purified CagXct samples labelled 3, 5, 9, 11, 13, 15 and 17 in (*a*). Lane *M* contains molecular-mass marker (labelled in kDa).

The cells were harvested by centrifugation (7000 rev min^−1^, 5 min), resuspended in a lysis buffer consisting of 50 m*M* Tris–HCl pH 7.0, 500 m*M* NaCl, 5%(*v*/*v*) glycerol, 1 m*M* phenylmethylsulfonyl fluoride (PMSF), 1%(*v*/*v*) Tween 20 and then sonicated on ice (Scientz-IID). The recombinant protein was purified from the supernatant by immobilized nickel-affinity column chromatography (GE Healthcare) and digested with TEV protease to remove the His tag and Trx tag (Fig. 1 ▸ *b*). The fractions were analyzed by SDS–PAGE (Fig. 1 ▸). The purified CagXct was then concentrated to 5 mg ml^−1^ and applied onto a size-exclusion chromatography column (HiLoad 16/600 Superdex 200 75 pg; GE Healthcare) in 25 m*M* Tris–HCl pH 7.0, 150 m*M* NaCl, 5% glycerol. The trace showed that most of the CagXct eluted in a monomeric form (Fig. 2 ▸ *a*). Fractions were assessed by 15% SDS–PAGE (Fig. 2 ▸ *b*).

The purified CagXct was concentrated and crystallized using the sitting-drop vapour-diffusion method in 96-well Intelli-Plates (Art Robbins Instruments). The crystallization trials were set up with an ARI robot (Art Robbins Instruments) using the following screens: Crystal Screen and Crystal Screen 2 (Hampton Research) and The PEGs, JCSG and Classics Suites (Qiagen). The drops had a total volume of 1 µl and consisted of a 1:1 ratio of protein solution to precipitant. The best crystals of CagXct grew from protein solution concentrated to 70 mg ml^−1^ with a precipitant consisting of 27% polyethylene glycol 4000, 100 m*M* HEPES pH 7.5, 10% 2-propanol.

### 2.3. Data collection and processing   

Data were collected at 100 K on the BL17U1 beamline of the Shanghai Synchrotron Radiation Facility (SSRF) at a wavelength of 0.9792 Å. The data were initially processed with *HKL*-2000 (Otwinowski & Minor, 1997 ▸) and the *CCP*4 suite (Winn *et al.*, 2011 ▸) in the orthorhombic space group *P*2_1_2_1_2_1_, with unit-cell parameters *a* = 33.1, *b* = 61.6, *c* = 48.4 Å, α = β = γ = 90°. Owing to problems in molecular repacement (MR) and refinement in this space group, the data were later reprocessed as triclinic using *DIALS* (Waterman *et al.*, 2016 ▸) in the *xia*2 pipeline (Winter *et al.*, 2013 ▸). The overall *DIALS* *R*_merge_ value decreased from 0.106 in *P*2_1_2_1_2_1_ to 0.050 in *P*1.

After the correct space group had been established by the downstream refinement as monoclinic *P*2_1_ with unique *b* = 61.6 Å, β = 90.23°, the data were reprocessed to 1.4 Å resolution in this space group with *XDS* (Kabsch, 2010 ▸) in the *xia*2 pipeline (Table 2 ▸).

### 2.4. Structure solution and refinement   

A promising MR solution was originally found in *P*2_1_2_1_2_1_ with the *MrBUMP*/*Phaser* pipeline (Keegan & Winn, 2008 ▸; McCoy *et al.*, 2007 ▸) using the structure of chain *B* (TraO) of the *E. coli* pKM101 plasmid-encoded outer membrane complex as a search model (Chandran *et al.*, 2009 ▸; PDB entry 3jqo; 19% sequence identity). The model, which contained one CagXct molecule per asymmetric unit (41% solvent), was refined using *REFMAC*5 (Murshudov *et al.*, 2011 ▸; Winn *et al.*, 2001 ▸) and rebuilt using *Coot* (Emsley *et al.*, 2010 ▸). Missing protein side chains were clearly visible in the electron density; however, the rebuilt structure could not be refined to an *R*_free_ value of below 0.38. An inspection of systematic absences proved to be inconclusive regarding the screw/rotation character of the crystal axes, therefore *MoRDa* (Vagin & Lebedev, 2015 ▸) was used to conduct MR and refinement in every possible orthorhombic space group. Surprisingly, high-scoring solutions were found in several space groups; however, the *P*2_1_2_1_2_1_ solution appeared to be the best since the *MoRDa* solutions in other space groups had higher *R* factors.

Intensity statistics (*L*-test; Padilla & Yeates, 2003 ▸) implemented in *POINTLESS* (Evans, 2011 ▸) suggested that the CagXct crystal belonged to a lower symmetry space group and was twinned. A distinct crystallographic dyad could not be determined by the processing statistics, since the merging *R* factors were close to 10% for each crystal axis.

To establish the true space group, the data were reprocessed in *P*1. The MR solution (four protein monomers) in this space group was found using *MoRDa* with PDB entry 3jqo chain *B* as the model. The solution had a *MoRDa* *Q*-factor of 0.71, a probability of correct solution of 99% and refined to an *R*_free_ of 0.40 without any model rebuilding, with most of the amino-acid side chains absent. The *CCP*4 program *Zanuda* (Lebedev & Isupov, 2014 ▸) was applied to the partially refined *P*1 model, which had an *R*_free_ of 0.32. The true space group, in which the model refined to the same *R* values, was monoclinic *P*2_1_, with unique *b* = 61.6 Å, β = 90.23°. The *R*_free_ values were 0.38 or higher in all other monoclinic and orthorhombic space groups. *SFCHECK* (Vaguine *et al.*, 1999 ▸) analysis of data reprocessed in *P*2_1_ clearly identified a nonmerohedral twinning operation (−*h*, −*k*, *l*) with an obliquity of 0.23° and a twinning fraction of 0.46. Twin isotropic *B*-factor refinement of the CagXct structure was performed with *REFMAC*. The atomic coordinates and structure factors for the CagXct structure have been deposited in the Protein Data Bank with accession code 5h3v.

## 3. Results and discussion   

### 3.1. Cloning, overexpression, purification and crystallization   

The soluble fragment (residues 396–498) of CagX was successfully cloned and expressed in *E. coli* strain BL21 (DE3) with a His tag and a Trx tag. The tags were removed by limited proteolysis of CagXct bound to a nickel immobilized metal ion-affinity column using TEV protease. The protein was further purified by a second run of nickel immobilized metal ion-affinity chromatography and size-exclusion chromatography. CagXct was concentrated to 70 mg ml^−1^ and crystallized by the vapour-diffusion method from PEG and 2-propanol.

### 3.2. Quality of the model   

**Figure 3:**
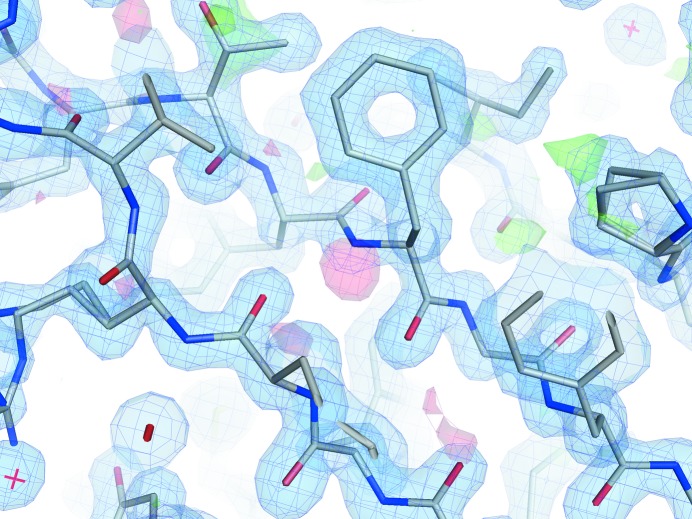
Electron-density maps around β-sheet 2 of CagXct monomer *A*. The 2*F*_o_ − *F*_c_ map (blue) is contoured at 1.4σ and the *F*_o_ − *F*_c_ map is contoured at 3.0σ (green) and −3.0σ (red). The difference density ripples (red and green) are likely to be owing to crystal twinning. This figure was prepared using *PyMOL* (Schrödinger).

The CagXct crystal structure was solved by MR and subjected to isotropic *B*-factor twin refinement in *REFMAC*5 at 1.4 Å resolution after the true space group had been established as monoclinic *P*2_1_ with a β angle close to 90°, with the crystal forming a nonmerohedral twin. The quality of the electron-density maps was mostly acceptable, although some ripples were observed in the *F*_o_ − *F*_c_ map calculated using the detwinned data (Fig. 3 ▸). These ripples are likely to be caused by crystal twinning since similar electron-density features have been reported for other twinned and order–disorder crystal structures (Lebedev *et al.*, 2006 ▸; Rye *et al.*, 2007 ▸). The CagXct model was refined to an *R* factor of 0.200 and an *R*_free_ of 0.249 in space group *P*2_1_ (Table 3 ▸). It contains two protein monomers with all residues built into the electron density, one PEG and two 2-propanol molecules and 129 waters. Many residue side chains were modelled with alternative conformations. The CagXct model contains no residues in the *cis*-conformation. Asp481 in both monomers of CagXct is a Ramachandran plot outlier, while Gly258 in the equivalent position in the TraO structure has similar main-chain torsion angles. The two monomers of CagXct comprising the asymmetric unit can be superimposed with an r.m.s.d. of 0.38 Å over all 103 C^α^ atoms.

### 3.3. Overall structure   

**Figure 4:**
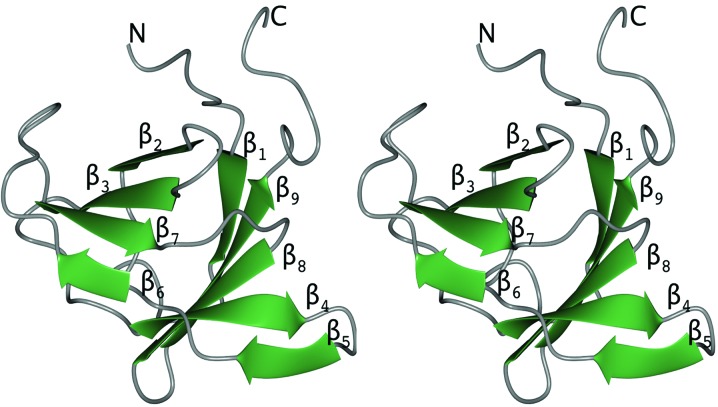
A stereo diagram showing a cartoon representation of CagXct, with the β-strands coloured green and loops coloured grey. The secondary-structure elements of this β-sandwich domain are numbered. Figs. 4 and 6 were prepared using *CCP*4*mg* (McNicholas *et al.*, 2011 ▸).

CagXct folds into a β-sandwich domain formed by two antiparallel β-sheets containing nine β-strands (Fig. 4 ▸). β-Sheet 1 contains strands β1, β4, β5, β8 and β9 and has Richardson topology 3, 1, −2x, −1 (Richardson, 1981 ▸). β-Sheet 2 contains strands β2, β3, β6 and β7 and has topology 1, 2x, −1.

The two independent CagXct monomers do not form any oligomer between themselves or with their crystal symmetry mates, which is in line with a monomer being the main species in solution, as can be seen from the size-exclusion chromatography trace (Fig. 2 ▸ *a*).

### 3.4. Comparison of CagXct with TraO   

A *DALI* search (Holm & Rosenström, 2010 ▸) reveals that the most similar structure to CagXct is the C-terminal domain of the VirB9 homologue TraO (TraOct) from the crystal structure of the pKM101 plasmid-encoded T4S outer membrane complex (Chandran *et al.*, 2009 ▸; PDB entry 3jqo; chain *B* was used as an MR model with a sequence identity of 19%). This ∼0.6 MDa complex represents a 14-fold rotational symmetry ring spanning the outer membrane which is formed by the pKM101 T4S system proteins TraOct, TraN and TraFct. An NMR structure is also available for TraO in complex with another component of the pKM101 T4S system, the VirB7-like TraN (PDB entry 3jqo; Chandran *et al.*, 2009 ▸).

**Figure 5:**
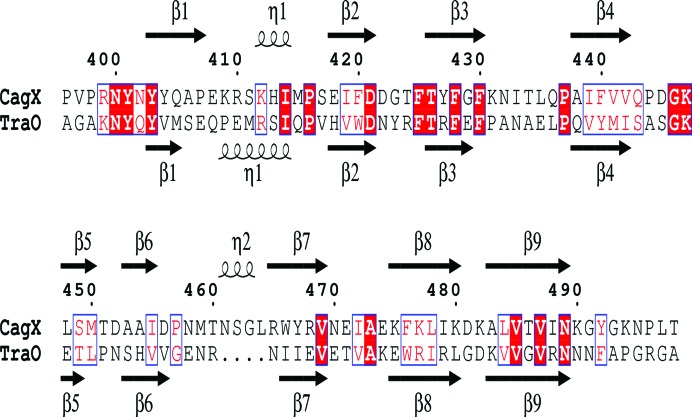
Anmino-acid sequence alignment of CagXct and TraOct. The secondary-structure elements are indicated above and below the alignment, respectively, as β-strands and η-helices (3_10_-helices). Conserved residues are shown in red boxes; matching amino acids with similar properties are shown in blue boxes. The secondary-structure assignments were carried out and the figure was produced using *ESPript*3 (Robert & Gouet, 2014 ▸).

CagXct and TraOct are remarkably similar, despite their low sequence identity (Figs. 5 ▸ and 6 ▸). The region 177–270 of the TraO monomer (PDB entry 3jqo; chain *B*) aligns with the CagXct monomer with an r.m.s.d. of 1.4 Å over 95% of the C^α^ atoms. Such a high level of structural similarity may explain the success of MR structure solution using the TraO model.

**Figure 6:**
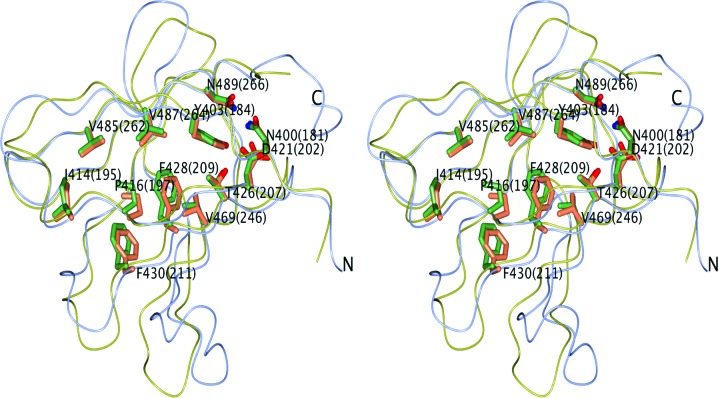
A stereo diagram showing the structural superposition of CagX (ice-blue worm model) and TraO (yellow; PDB entry 3jqo). Side chains of residues forming the conserved protein core are shown with C atoms in coral (CagX) or green (TraO). Residue numbers of TraO are given in parentheses after these of the CagX residues.

To maintain structural similarity, many buried protein residues which form the protein core of TraO are conserved or substituted by residues with similar properties in CagX. Remarkably, most of these conserved residues retain the same side-chain conformation (Fig. 6 ▸). Generally, it would appear that the conservation of the overall fold/shape of the VirB9-like domain is more important for the function of this member of the T4S system than the preservation of specific individual residues that may be involved in interactions with other proteins forming the outer membrane complex of the T4S system.

### 3.5. Conservation of protein–protein interactions in T4S   

The structure of the outer membrane complex of the T4S plasmid pKM101 (Chandran *et al.*, 2009 ▸) provides insight into the general architecture of bacterial T4S systems. A TraOct domain in this ring structure interacts with two neighbouring TraO monomers, two TraF monomers and a single TraN monomer. Since relatively little is known about the inter­actions of CagX with its partners in the *H. pylori* T4S system, it is interesting to map the sequence/structure conservation features between TraO and CagXct onto these monomer–monomer interactions.

TraN is a long peptide which winds around TraO. It adds an additional β-strand to β-sheet 1 of TraO, which is observed in both the binary complex TraO–TraN and in the heterotrimeric outer membrane complex. Most amino-acid residues of strand β1, which runs antiparallel to TraN in TraO, are not conserved in CagX; however, the main chains of the matching residues of both these T4S proteins have the same conformation and solvent accessibility. Thus, it appears likely that the β-sheet interaction between TraO and TraN is reproduced in the Cag–CagT interface.

Interactions between different TraO monomers in the 14-fold ring of the outer membrane complex of T4S plasmid pKM101 are not extensive and the residues involved in these interactions do not appear to be conserved in CagX.

Interactions between TraO and the two monomers of TraF in this complex are more extensive; however, there is little conservation of amino-acid residues in this interface. Interestingly, one of the 2-propanol molecules binds to the main-chain N atom of the Ramachandran plot outlier Asp481 in monomer *A* in the CagXct structure. The main-chain N atom of the equivalent Gly258 in TraO forms a hydrogen bond to the main-chain O atom of Gly364 in TraF, with the positions of Gly364 and the 2-propanol ligand O atoms overlapping. This may suggest the preservation of another main-chain inter­action in the CagX–CagY interface.

**Figure 7:**
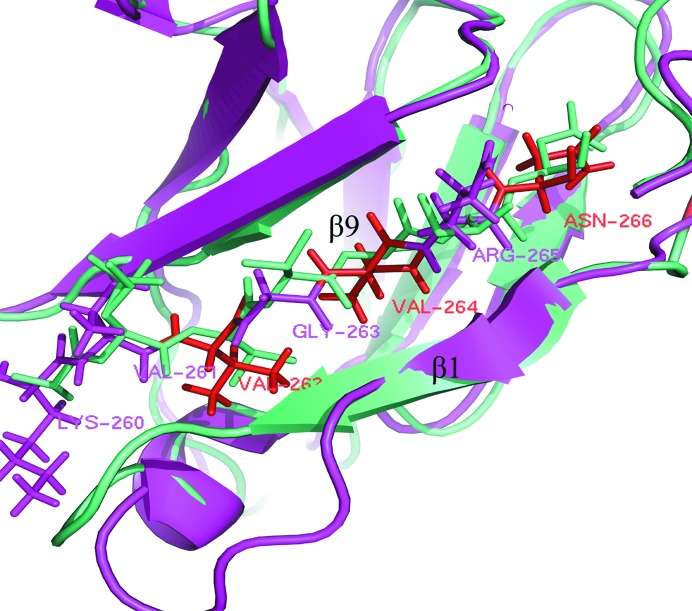
Comparison of the protein-binding region of the β9 strand (shown as sticks; β1 strands are shown as cartoons) in CagXct (cyan) and in TraO (magenta, only the residues of TraO are labelled). Apart from the binding residues, there is a high sequence-similarity pattern: ‘*x*V*x*V*x*N’ (these residues are shown in red; *x* represents the binding amino-acid residues).

The sequence pattern ‘*x*V*x*V*x*N’ (where *x* represents a binding residue) is shared by both structures in the β9 strand in TraO and CagXct, suggesting that the conserved non­binding residues in this pattern may play a role in maintaining the proper distance with the β1 strand in a spatial configuration to ensure binding to a protein partner (Fig. 7 ▸). However, the binding residues are not conserved in β9; Leu484, Thr486 and Ile488 in CagXct correspond to Val261, Gly263 and Arg264 in TraO (Figs. 5 ▸ and 7 ▸). The diversity in binding residues should be determined by their different interacting protein substrates.

CagXct is only the second VirB9 homologue for which a three-dimensional structure has been elucidated. Its structure will provide additional insight into binding and translocation mechanisms of the transmembrane core complex in *H. pylori* and other T4S systems.

## Supplementary Material

PDB reference: CagX, 5h3v

## Figures and Tables

**Table 1:** Cloning of expression constructs

Source organism	*H. pylori*
DNA source	cDNA isolated from *H. pylori* 26695
Forward primer (FCagXct-EcoRI)	CCGGAATTCCCCGTGCCTAGAAACTACAACTAC
Reverse primer (NCagXct-XhoI)	CCGCTCGAGTTATGTCAATGGATTTTTCCCATAGCC
Cloning vector	pET-32a
Expression vector	pET-32a
Expression host	*E. coli* BL21 (DE3)
Complete amino-acid sequence of the construct product	PVPRNYNYYQAPEKRSKHIMPSEIFDDGTFTYFGFKNITLQPAIFVVQPDGKLSMTDAAIDPNMTNSGLRWYRVNEIAEKFKLIKDKALVTVINKGYGKNPLT

**Table 2:** Data collection and processing Values in parentheses are for the outer shell.

Diffraction source	BL17U1, SSRF
Wavelength (Å)	0.97946
Temperature (K)	100
Detector	ADSC Q315R
Rotation range per image (°)	1
Total rotation range (°)	180
Exposure time per image (s)	0.8
Space group	*P*2_1_
*a*, *b*, *c* (Å)	33.1, 61.6, 48.4
α, β, γ (°)	90.00, 90.23, 90.00
Resolution range (Å)	38.07–1.40 (1.44–1.40)
Total No. of reflections	130565
No. of unique reflections	34978
Completeness (%)	91.7 (56.0)
〈*I*/σ(*I*)〉	12.3 (3.0)
*R*_merge_ (%)†	5.0 (34.7)
Multiplicity	3.7 (3.4)
CC_1/2_	0.997 (0.803)

† *R*_merge_ = 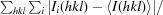 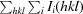, where *I*_*i*_(*hkl*) are the intensities of the individual measurements of a given reflection *hkl* and 〈*I*(*hkl*)〉 is the average intensity over all replicates of that reflection, 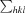 is the sum over all reflections and 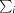 is the sum over *i* measurements of the reflection.

**Table 3:** Structure solution and refinement of CagXct

PDB code	5h3v
Resolution range (Å)	38.07–1.40 (1.43–1.40)
Final *R*_cryst_	0.200 (0.291)
Final *R*_free_	0.249 (0.442)
No. of twin domains	2
Twin fractions	0.509 (*h*, *k*, *l*), 0.491 (−*h*, −*k*, *l*)
*L*-test for twinning	〈|*L*|〉 = 0.40, 〈*L* ^2^〉 = 0.22
R.m.s. deviations	
Bonds (Å)	0.012
Angles (°)	1.712
Wilson *B* factor (Å^2^)†	20.2
No. of protein residues	206
No. of solvent atoms	145
Average *B*-factor values	
Protein (Å^2^)	15.0
Solvent (Å^2)^	22.6
Ramachandran plot analysis‡	
Most favoured (%)	85.2
Additionally allowed (%)	13.6
Generously allowed (%)	0.6
Disallowed	0.6

† The Wilson *B* factor was calculated using *SFCHECK* (Vaguine *et al.*, 1999 ▸).

‡ The Ramachandran plot analysis was performed by *PROCHECK* (Laskowski *et al.*, 1993 ▸).
